# Reply: Is serum matrix metalloproteinase 9 a useful biomarker in detection of colorectal cancer? Considering pre-analytical interference that may influence diagnostic accuracy

**DOI:** 10.1038/sj.bjc.6604492

**Published:** 2008-07-22

**Authors:** N G Hurst

**Affiliations:** 1 Department of Surgery, Derby City General Hospital, Uttoxeter Road, Derby DE22 3NE, UK

**Sir**,

We thank Professor Jung for his interest in our work and congratulate him on both his previous published work and his more recent small demonstrative study.

As he correctly states, serum analysis was employed, without clot activators, for our own pilot study, initiated before his and other authors' publications on choice of substrate. Technical details in minutiae form were not included in the final submitted paper for reasons of brevity and space constraints. However, it is almost universally agreed that, without similar space constraints, further details, particularly pertaining to pre-study planning, serum acquisition and post-venesection processing would be useful. Furthermore, description of quality control measures and internal control methods would be useful adjuncts for those wishing to replicate work done.

However, Professor Jung may have overlooked the underlying premise and methodology of our study in that this represented a comparative analysis of total detectable levels of serum MMP9, collected under tightly controlled protocols, for the prediction of the presence of significant colorectal pathology.

The absolute levels obtained were less critical in this study than the relative levels seen within the population measured, harbouring colorectal neoplasia or otherwise. No assertion is made that the absolute serum MMP9 levels are applicable to the general population as a whole, nor cohorts in different regions/countries.

In addition, choice of assay employed in measurement has significant effects on the absolute levels reported, whether total/active/proenzyme or complexed protein is measured. Once again, in a study design as employed in our pilot study, relative levels within a population providing its own controls are more useful than imported levels obtained under potentially varied conditions elsewhere.

Professor Jung's final comments regarding sample type of choice are noted with appreciation, and further discussion will no doubt ensue within the research group.

